# Cobalt- and rhodium-catalyzed carboxylation using carbon dioxide as the C1 source

**DOI:** 10.3762/bjoc.14.221

**Published:** 2018-09-19

**Authors:** Tetsuaki Fujihara, Yasushi Tsuji

**Affiliations:** 1 Department of Energy and Hydrocarbon Chemistry, Graduate School of Engineering, Kyoto University, Nishikyo-ku, Kyoto 615-8510, Japan

**Keywords:** carbon dioxide, carboxylation, cobalt, homogeneous catalysts, rhodium

## Abstract

Carbon dioxide (CO_2_) is one of the most important materials as renewable chemical feedstock. In this review, the Co- and Rh-catalyzed transformation of CO_2_ via carbon–carbon bond-forming reactions is summarized. Combinations of metals (cobalt or rhodium), substrates, and reducing agents realize efficient carboxylation reactions using CO_2_. The carboxylation of propargyl acetates and alkenyl triflates using cobalt complexes as well as the cobalt-catalyzed reductive carboxylation of α,β-unsaturated nitriles and carboxyamides in the presence of Et_2_Zn proceed. A Co complex has been demonstrated to act as an efficient catalyst in the carboxylation of allylic C(sp^3^)–H bonds. Employing zinc as the reductant, carboxyzincation and the four-component coupling reaction between alkyne, acrylates, CO_2_, and zinc occur efficiently. Rh complexes also catalyze the carboxylation of arylboronic esters, C(sp^2^)–H carboxylation of aromatic compounds, and hydrocarboxylation of styrene derivatives. The Rh-catalyzed [2 + 2 + 2] cycloaddition of diynes and CO_2_ proceeds to afford pyrones.

## Introduction

Carbon dioxide (CO_2_) is one of the most important materials as renewable feedstock [1–4]. However, the thermodynamic and kinetic stability of CO_2_ sometimes limits its utility. Classically, harsh reaction conditions such as high temperature and high pressure of CO_2_ were required. To overcome these problems, the use of transition-metal catalysts has been considered as a fundamental and reliable method. In the last decade, considerable attention has been focused on the development of the catalytic fixation of CO_2_ via carbon–carbon (C–C) bond formation using a variety of organic compounds as starting materials [5–20]. A key factor for the successful catalytic fixation of CO_2_ is the carbon–metal bond formation when transition metals are used as the catalyst. In addition, the choice of suitable reducing agents is also crucial for realizing effective carboxylation reactions.

In this review, the Co- and Rh-catalyzed transformations of CO_2_ via C–C bond-forming reactions are summarized. First, we describe Co-catalyzed carboxylation reactions, including the carboxylation of propargyl acetates and alkenyl triflates. Then, the Co-catalyzed reductive carboxylation of α,β-unsaturated nitriles and carboxyamides is addressed. In addition, a Co catalyst can catalyze the allylic C(sp^3^)–H carboxylation of allylarenes when a suitable ligand is used. In the presence of zinc powder, the Co-catalyzed carboxyzincation of alkynes and the four-component coupling reaction between alkyne, acrylates, CO_2_, and zinc proceed in an efficient manner. Visible-light-driven hydrocarboxylation reactions are shown. We also summarize carboxylation reactions catalyzed by rhodium that is a homologous element of cobalt. Carboxylations of arylboronic esters are described. Rh complexes are also effective catalysts in C(sp^2^)–H carboxylation reactions. Employing Et_2_Zn or visible light, the Rh-catalyzed hydrocarboxylation of styrene derivatives has been achieved. Furthermore, the formation of pyrones from diynes and CO_2_ can be effectively catalyzed by Rh complexes.

## Review

### Cobalt catalysts

#### Carboxylation of propargyl acetates

Allyl and propargyl electrophiles, such as halides and esters, are well known as efficient reagents in transition-metal-catalyzed C–C bond-forming reactions [21–23]. In particular, the carboxylation of allyl esters with CO_2_ has been catalyzed by Pd or Ni under electrochemical reaction conditions [24–25]. For catalytic reactions using reducing agents, Martin reported Ni-catalyzed regiodivergent carboxylation of allyl acetates in the presence of Mn as the reductant [26]. Mita and Sato found that Pd-catalyzed carboxylation of allylic alcohols proceeded using Et_2_Zn as the reducing agent [27]. The carboxylation of propargyl chloride was reported as one of the examples concerning the Ni-catalyzed carboxylation of benzyl chlorides [28].

**Scheme 1:**
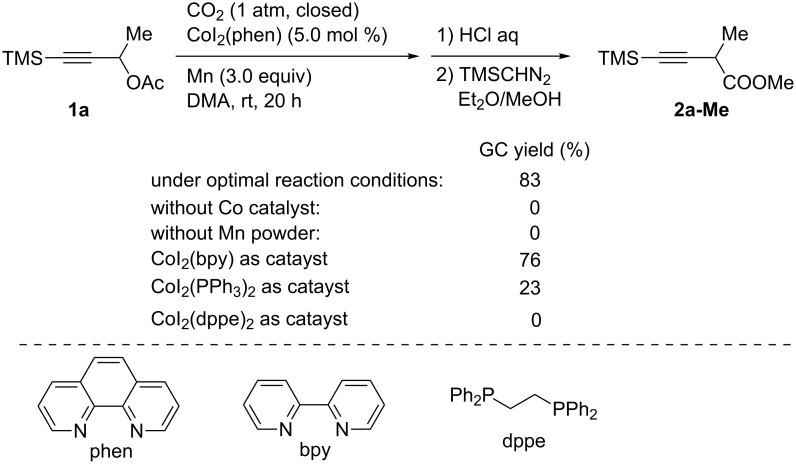
Optimization of the Co-catalyzed carboxylation of **1a**.

We have found that Co complexes can catalyze the carboxylation of propargyl acetates with CO_2_ using Mn powder as the reducing agent [29]. Thus, the carboxylation of a propargyl acetate **1a** was performed in the presence of CoI_2_(phen) (phen = 1,10-phenanthroline) and Mn powder (3.0 equiv) in *N*,*N*-dimethylacetamide (DMA) under an atmospheric pressure of CO_2_ at room temperature (Scheme 1). Under optimized reaction conditions, the carboxylated product **2a-Me** was obtained in 83% yield after derivatization to the corresponding methyl ester. In the absence of the Co catalyst, **2a-Me** was not obtained. Moreover, Mn powder proved to be essential for the carboxylation to proceed. Using CoI_2_(bpy) (bpy = 2,2′-bipyridine) as the catalyst afforded **2a-Me** in 76% yield, whereas CoI_2_(PPh_3_)_2_ and CoI_2_(dppe) (dppe = 1,2-bis(diphenylphosphino)ethane) suppressed the carboxylation.

**Scheme 2:**
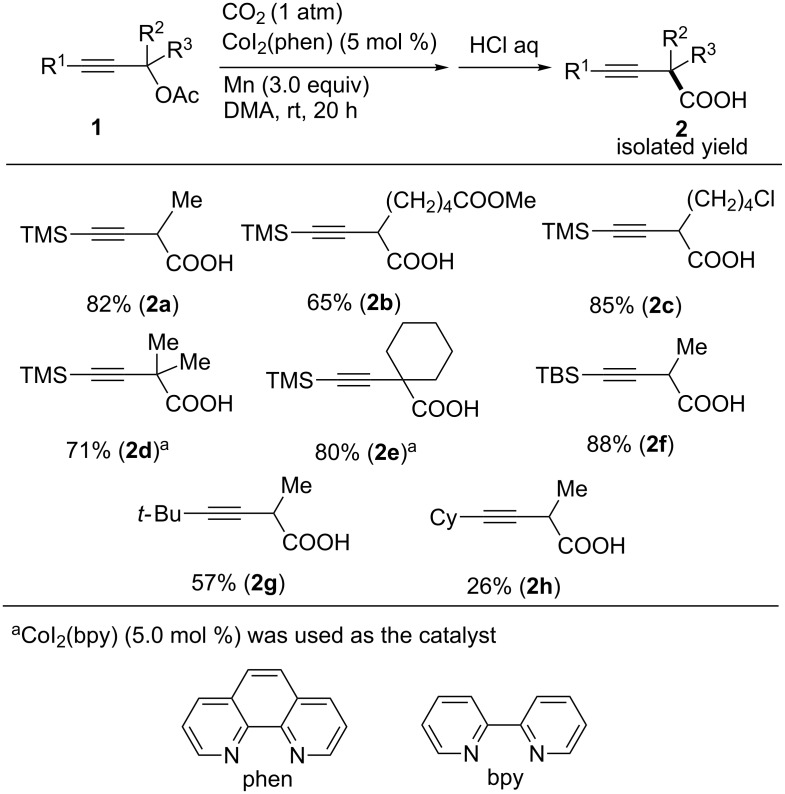
Co-catalyzed carboxylation of propargyl acetates **1**.

The carboxylation of various propargyl acetates containing the trimethylsilyl (TMS) group as the R^1^ group proceeded under the optimal reaction conditions, affording the corresponding carboxylic acids **2b**–**e** in good-to-high yields (Scheme 2). Notably, the ester and chloro functionalities in **2b** and **2c**, respectively, were compatible with the reaction conditions. For the carboxylation of tertiary-alcohol-derived acetates to the corresponding carboxylic acids **2d**,**e**, CoI_2_(bpy) was found to be an effective catalyst. The yields of product **2** decreased when less bulky substituents (R^1^) were used. Thus, **1f** (R^1^ = *tert*-butyldimethylsilyl) afforded the corresponding product **2f** in 88% yield, whereas **1g** (R^1^ = *t-*Bu) and **1h** (R^1^ = Cy) afforded **2g** and **2h** in 57% and 26% yields, respectively.

**Scheme 3:**
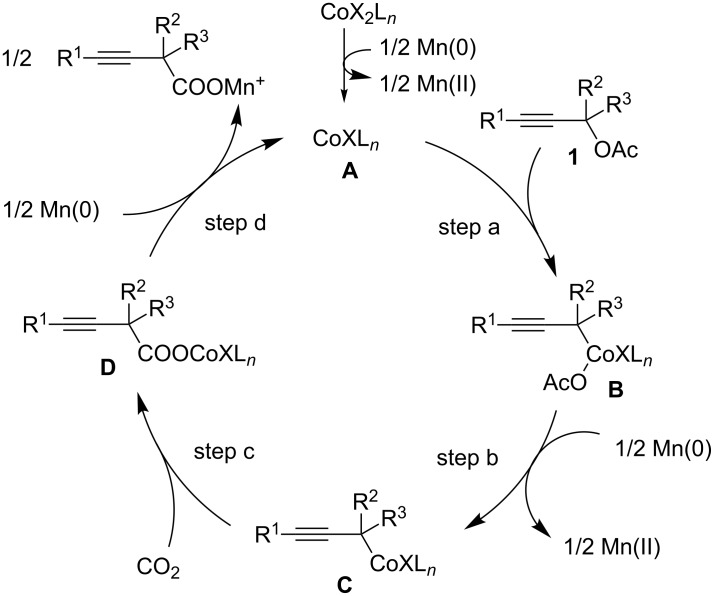
Plausible reaction mechanism for the Co-catalyzed carboxylation of propargyl acetates **1**.

Scheme 3 presents a plausible reaction mechanism for this transformation. Accordingly, a Co(I) catalytic species **A** is first generated by the reduction of Co(II) with Mn. Secondly, the oxidative addition of the C–O bond in **1** occurs, affording Co(III) intermediate **B** (step a) [30]. Next, the propargyl Co(III) species **B** is reduced by Mn, producing the corresponding propargyl Co(II) intermediate **C** (step b). Subsequently, the nucleophilic Co species **C** reacts with CO_2_, which provides carboxylate Co(II) intermediate **D** (step c). Finally, the reduction of **D** with Mn affords the corresponding carboxylate, regenerating the Co(I) catalytic species **A** (step d).

#### Carboxylation of alkenyl and aryl triflates

The catalytic carboxylation of aryl halides and pseudohalides using CO_2_ is an important reaction to yield benzoic acid derivatives. In 2009, Martin reported the Pd-catalyzed carboxylation of aryl bromides using ZnEt_2_ as the reductant [31]. In 2012, we first reported the Ni-catalyzed carboxylation of aryl chlorides and vinyl chlorides using Mn powder as the suitable reductant [32]. These reactions can be performed under mild conditions, i.e., an atmospheric pressure of CO_2_ at room temperature.

**Scheme 4:**
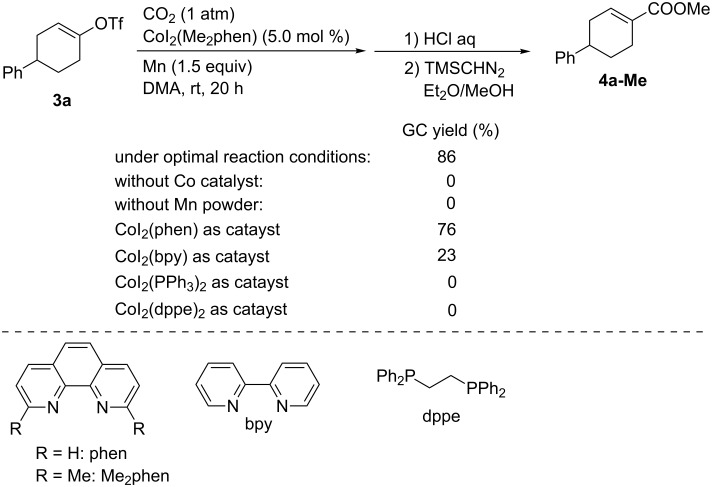
Optimization of the Co-catalyzed carboxylation of **3a**.

We also reported the Co-catalyzed carboxylation of alkenyl and aryl trifluoromethanesulfonates (triflates) as substrates [33]. As a model reaction (Scheme 4), alkenyl triflate **3a** was selected as the substrate, and the carboxylation of **3a** was performed using Mn powder (1.5 equiv) as the reductant in DMA as the solvent under an atmospheric pressure of CO_2_ at room temperature. Employing CoI_2_(Me_2_phen) (Me_2_phen = 2,9-dimethyl-1,10-phenanthroline) as the catalyst, **4a-Me** was obtained in 86% yield after esterification. Other bidentate ligands such as bpy, phen, and dppe were not suitable for this reaction. Control experiments revealed that both the Co catalyst and the Mn reductant were indispensable to the reaction.

**Scheme 5:**
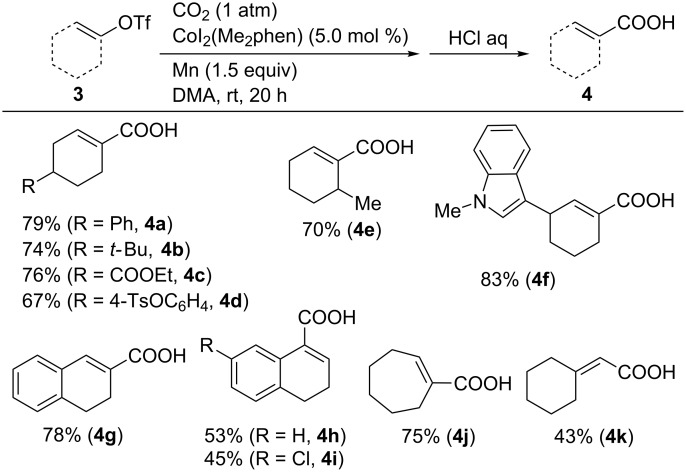
Co-catalyzed carboxylation of vinyl triflates **3**.

The carboxylation of diverse alkenyl triflates was also examined. As a result, the desired carboxylic acids **4a**–**k** were obtained in good-to-high yields, as shown in Scheme 5. Notably, the ester and *p*-toluenesulfonate functionalities in **4c** and **4d**, respectively, were tolerated. An indole-functionalized substrate **3f** was converted into its corresponding carboxylic acid **4f**. Conjugated alkenyl triflates **3g–i** were also subjected to the reaction, and the desired carboxylic acids **4g–i** were obtained in moderate-to-high yields. Furthermore, the seven-membered cyclic substrate **3j** that was derived from cycloheptanone afforded its corresponding conjugated carboxylic acid **4j** in 75% yield. The alkenyl triflate **3k** prepared from the corresponding aldehyde was also carboxylated, and its corresponding product **4k** was obtained in moderate yield.

**Scheme 6:**
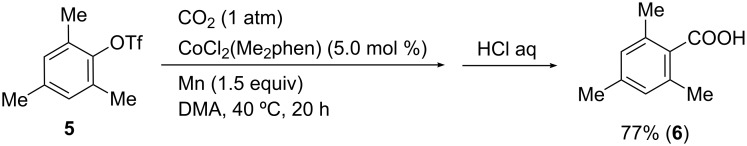
Co-catalyzed carboxylation of a sterically hindered aryl triflate **5**.

The catalyst CoI_2_(Me_2_phen) was also effective with sterically hindered aryl triflates: the carboxylation of mesityl triflate (**5**) at 40 °C proceeded successfully, affording **6** in 77% yield (Scheme 6)

#### Carboxylation of α,β-unsaturated nitriles and esters

α,β-Unsaturated carbonyl compounds are good substrates for conjugate additions that use a catalytic amount of a metal complex and a stoichiometric amount of reductant, as exemplified by the reductive aldol reaction of α,β-unsaturated nitriles catalyzed by cobalt using phenylsilane as the reductant [34].

**Scheme 7:**
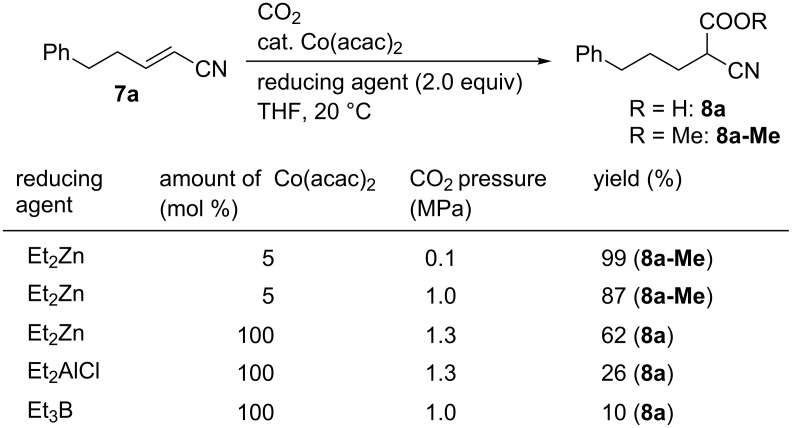
Optimization of the Co-catalyzed carboxylation of **7a**.

Yamada found that the reductive carboxylation of α,β-unsaturated compounds with CO_2_ proceeded in the presence of Co catalysts and reductants (Scheme 7) [35–36]. When the reaction of 5-phenylpent-2-enenitrile (**7a**) was performed in the presence of catalytic Co(acac)_2_ and with Et_2_Zn as the reductant, the carboxylation proceeded to yield **8a-Me** after its derivatization to the corresponding methyl ester. In these reactions, the selection of the reductant is crucial: other reductants such as Et_2_AlCl and Et_3_B yielded the corresponding product in low yields even after using a stoichiometric amount of Co(acac)_2_ at high pressure.

**Scheme 8:**
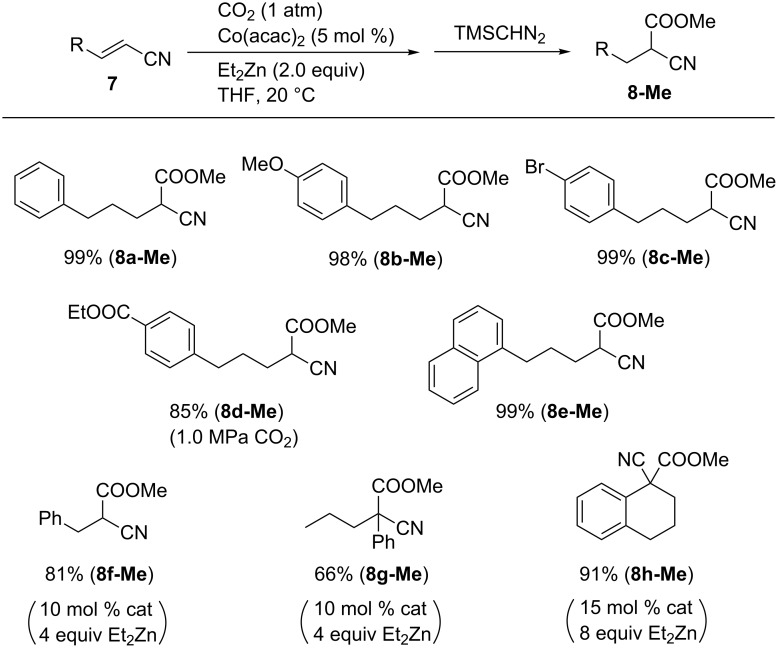
Scope of the reductive carboxylation of α,β-unsaturated nitriles **7**.

Under the optimal reaction conditions with the Co(acac)_2_/Et_2_Zn system, various α,β-unsaturated nitriles were carboxylated to the corresponding products, which were isolated as methyl esters (Scheme 8). Thus, compound **7** bearing alkyl, ether, ester, and halide substituents exhibited good reactivity. Cinnamonitrile afforded **8f-Me** in 81% yield using 10 mol % of catalyst. α-Phenyl-substituted α,β-unsaturated nitrile also reacted with CO_2_, affording the carboxylated product **8g-Me** in good yield. In addition, compound **8h-Me** was obtained from the reaction of α-cyano-substituted dihydronaphthalene **7h** with CO_2_ in the presence of 15 mol % of catalyst and 8 equiv of Et_2_Zn.

**Scheme 9:**
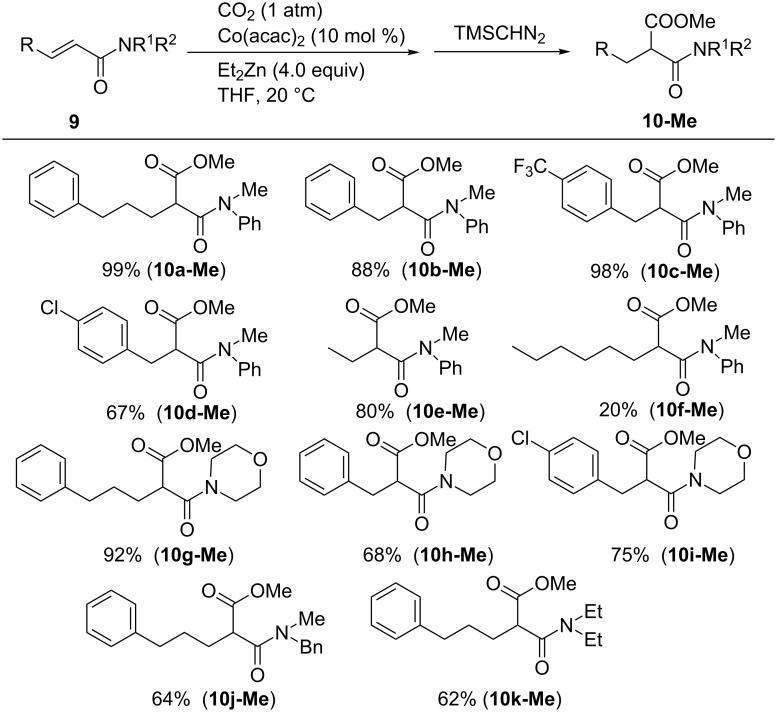
Scope of the carboxylation of α,β-unsaturated carboxamides **9**.

The Co(acac)_2_/Et_2_Zn system can also be applied to carboxylate α,β-unsaturated carboxamides **9** (Scheme 9). By using 10 mol % of Co(acac)_2_ and 4 equiv of Et_2_Zn, *N*-methylanilide derivatives **9a**–**f** were smoothly converted into the corresponding products **10a–f** in high yields. Trifluoromethyl and chloro substituents were tolerated in these reactions, judging by the formation of products **10c-Me** and **10d-Me**. With regard to other amide groups, morpholides **9g–i** could be used and benzylmethylamide- and diethylamide-bearing substrates, which afforded the corresponding products **10j-Me** and **10k-Me**, albeit with moderate yields.

#### Allylic C(sp^3^)–H bond carboxylation

**Scheme 10:**
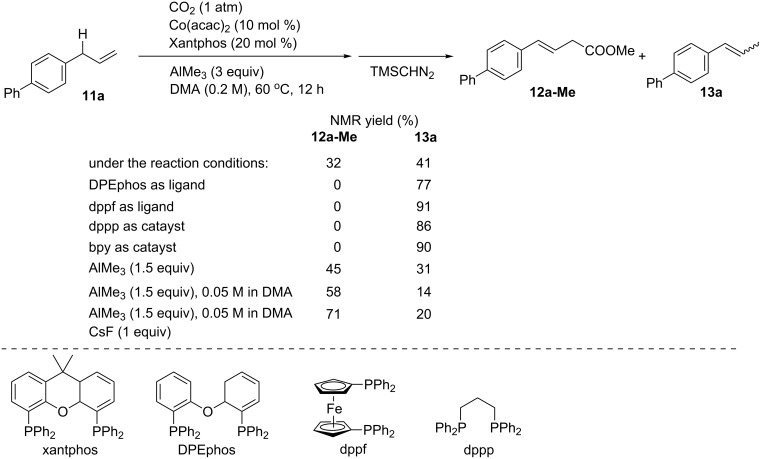
Optimization of the Co-catalyzed carboxylation of **11a**.

The development of methods for the catalytic carboxylation of less reactive C–H bonds with CO_2_ is crucial regarding both C–H activation and CO_2_ ﬁxation processes. Mita and Sato reported a cobalt-catalyzed allylic C–H carboxylation of allylarenes (Scheme 10), in which 1-allyl-4-phenylbenzene (**11a**) was reacted with CO_2_ (1 atm) in the presence of AlMe_3_ (3 equiv) using catalytic amounts of a Co precursor and ligands [37]. The catalytic system comprising Co(acac)_2_ and Xantphos (4,5-bis(diphenylphosphino)-9,9-dimethylxanthene) afforded the corresponding carboxylated product **12a-Me** in an NMR yield of 71% after CH_2_N_2_ treatment. In the reaction mixture, oleﬁn isomers were also generated in 20% yield. The ligands were found to have a strong influence in yields and selectivity. Thus, the use of DPEphos (2,2′-bis(diphenylphosphino)diphenyl ether), dppf (1,1′-bis(diphenylphosphino)ferrocene), dppp (1,3-bis(diphenylphosphino)propane), and bpy as ligands afforded the oleﬁn isomerization product as the major product. Further screening of the reaction conditions revealed that the amount of AlMe_3_ was critical: the product yield increased with decreasing AlMe_3_ to 1.5 equiv. The concentration of **11a** also affected the efficiency of the reaction, and the isomerization of oleﬁns could be suppressed at lower concentrations of **11a**, affording the desired **12a-Me** in 58% yield. With the addition of 1 equiv of CsF, the carboxylation was further accelerated to give **12a-Me** in 71% yield.

**Scheme 11:**
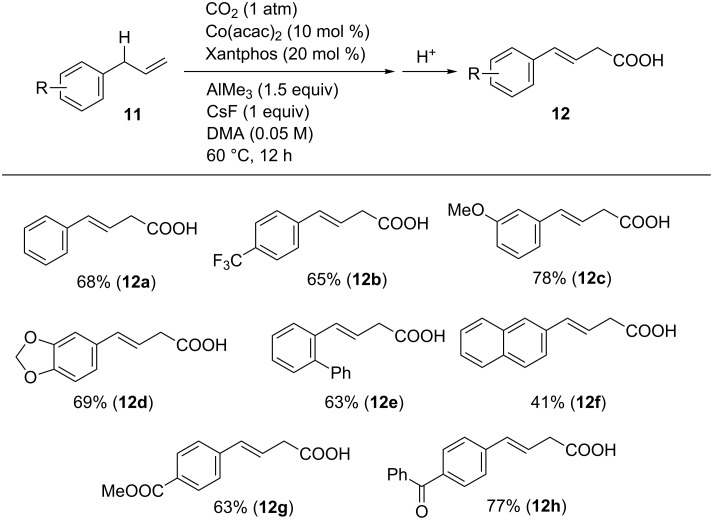
Scope of the carboxylation of allylarenes **11**.

Using optimized reaction conditions, the substrate scope was examined (Scheme 11). Allylbenzene was converted into its corresponding carboxylated product **12a** in an isolated 68% yield, and various functionalized allylarenes bearing triﬂuoromethyl (**11b**) and alkoxy (**11c**,**d**) substituents were tolerated. The selectivity of the reaction was demonstrated with substrates **11g** and **11h** containing ester and ketone moieties, respectively, which are generally more reactive toward nucleophiles than CO_2_.

**Scheme 12:**
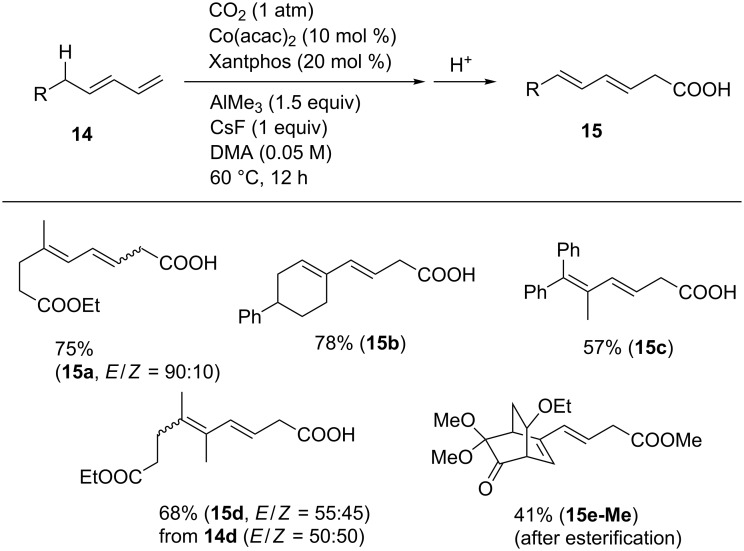
Scope of the carboxylation of 1,4-diene derivatives **14**.

Notably, the Co-catalyst system was found to be applicable for the carboxylation of 1,3-diene derivatives **14** with CO_2_ (Scheme 12), which afforded various hexa-3,5-dienoic acid derivatives. Diene **14a** was converted into the corresponding carboxylic acid **15a** in good yield. In addition, 1,4-dienes having cyclohexenyl and geminal diphenyl substituents (**14b** and **14c**) produced their corresponding linear carboxylic acids **15b** and **15c** in 78% and 57% yields, respectively. Substrate **14e** containing a bicyclo[2.2.2]octane framework with ketone and dimethyl ketal moieties was also converted and the corresponding product was isolated as the methyl ester **15e-Me** after esterification.

**Scheme 13:**
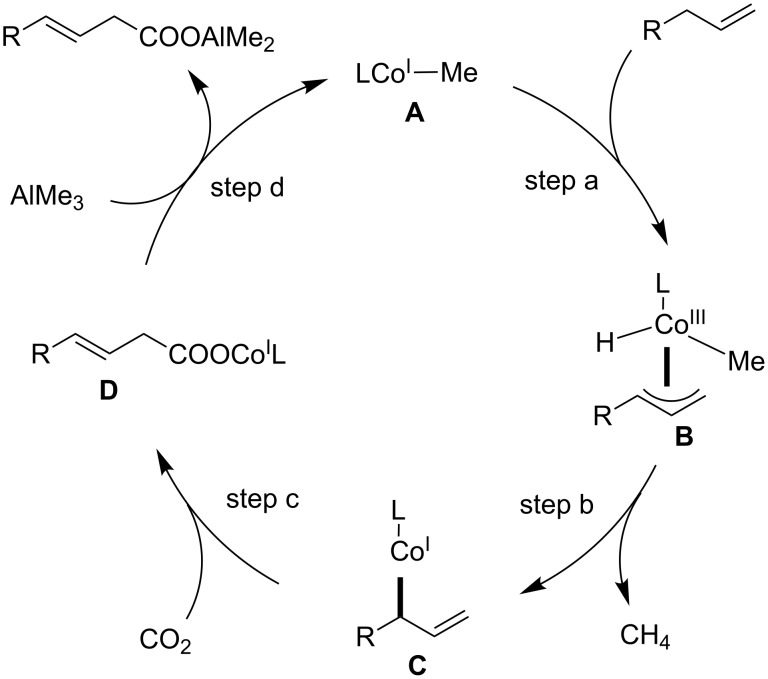
Plausible reaction mechanism for the Co-catalyzed C(sp^3^)–H carboxylation of allylarenes.

For the Co-catalyzed C(sp^3^)–H carboxylation of allylarenes, a mechanism shown in Scheme 13 can be envisaged. The process starts with the generation of a low-valent methyl-Co(I) species **A** by the reaction of the Co(II) complex with AlMe_3_. The C–C double bond of the substrate then coordinates to the metal, and the subsequent cleavage of the adjacent allylic C–H bond affords η^3^-allyl-Co(III) species **B** (step a). Subsequently, the reductive elimination of methane from **B** yields the low-valent allyl-Co(I) species **C** (step b). Then, C–C bond formation at the γ-position occurs via a reaction with CO_2_, affording the carboxylate Co species **D** (step c). Finally, a linear carboxylated product is obtained by the transmetalation between **D** and AlMe_3_, with the concomitant regeneration of methyl-Co(I) **A** (step d).

#### Carboxyzincation of alkynes

The good reactivity and high functional-group compatibility of organozinc compounds render them as important reagents in organic synthesis [38–39]. For their preparation, direct and useful methods such as the transition-metal-catalyzed carbozincation of alkynes that affords stereodefined alkenylzinc compounds have been developed. To date, a variety of organozinc reagents (RZnX and R_2_Zn: R = aryl, alkyl, alkenyl, alkynyl, allyl, and benzyl groups) have been used in these reactions, and the corresponding alkenylzinc compounds can be prepared.

**Scheme 14:**
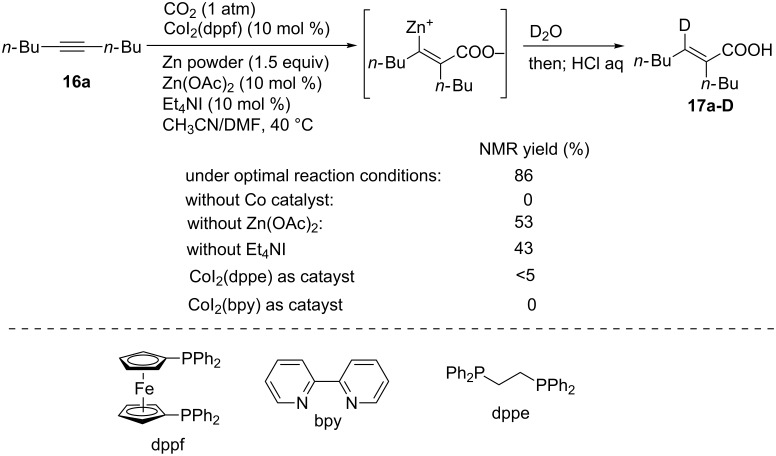
Optimization of the Co-catalyzed carboxyzincation of **16a**.

In this context, we reported the first carboxyzincation of alkynes using CO_2_ and Zn metal powder in the presence of a cobalt complex as the catalyst (Scheme 14) [40]. 5-Decyne (**16a**) was treated with Zn powder (1.5 equiv) in the presence of CoI_2_(dppf) (10 mol %), Zn(OAc)_2_ (10 mol %), and Et_4_NI (10 mol %) in a mixture of CH_3_CN and DMF (v/v = 10:1) under an atmospheric pressure of CO_2_ at 40 °C. When the reaction mixture was quenched with D_2_O (>99% D), deuterated **17a-D** was obtained in a ^1^H NMR yield of 80% with excellent deuterium incorporation ratio (94%) at the β-position. Although Zn(OAc)_2_ and Et_4_NI were not indispensable for the reaction to proceed, these two additives caused an increased product yield. In contrast, the reaction did not occur in the absence of the catalyst. The use of the dppf ligand also proved to be essential, because other ligands such as dppe and bpy were not effective in the reaction.

**Scheme 15:**
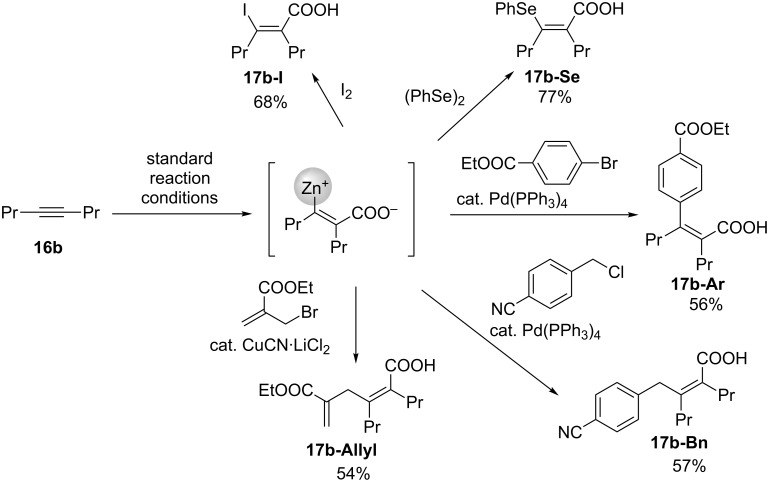
Derivatization of the carboxyzincated product.

After the reaction with 4-octyne (**17b**) under the aforementioned conditions, the reactions with I_2_ and (PhSe)_2_ produced **17b-I** and **17b-Se** in good yields (Scheme 15). Notably, **16b** was successfully subjected to the Pd-catalyzed Negishi coupling with aryl bromide, affording the corresponding sterically congested alkene **17b-Ar** in 56% yield after two steps. The Negishi coupling with benzyl chloride and the Cu-catalyzed allylation of allyl bromide also afforded the corresponding products **17b-Bn** and **17b-Allyl**, respectively, in good yields.

**Scheme 16:**
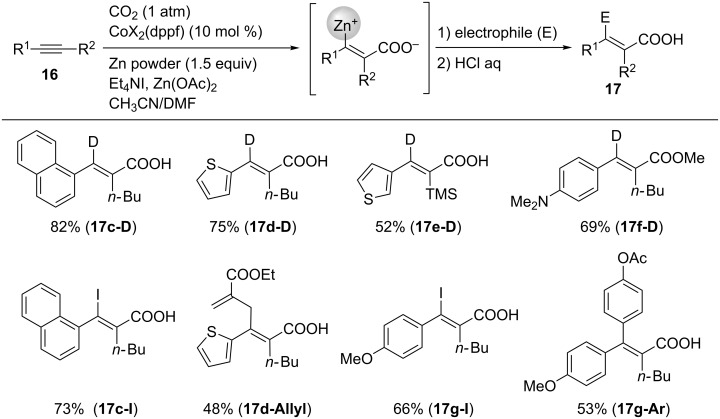
Co-catalyzed carboxyzincation of alkynes **16**.

The reaction successfully proceeded even with unsymmetrical internal alkynes. For instance, 1-(1-naphthyl)-1-hexyne (**16c**) afforded **17c-D** in 82% yield with excellent regioselectivity (Scheme 16). Thienyl-substituted alkynes such as **16d** and **16e** selectively furnished **17d-D**, **17d-Allyl**, and **17e-D**. Unsymmetrical internal alkynes bearing 4-Me_2_NC_6_H_4_ and 4-MeOC_6_H_4_ moieties (**16f** and **16g**) afforded **17f-D**, **17g-D**, and **17g-Ar** regioselectively after treatment with D_2_O or aryl iodide/Pd catalyst.

**Scheme 17:**
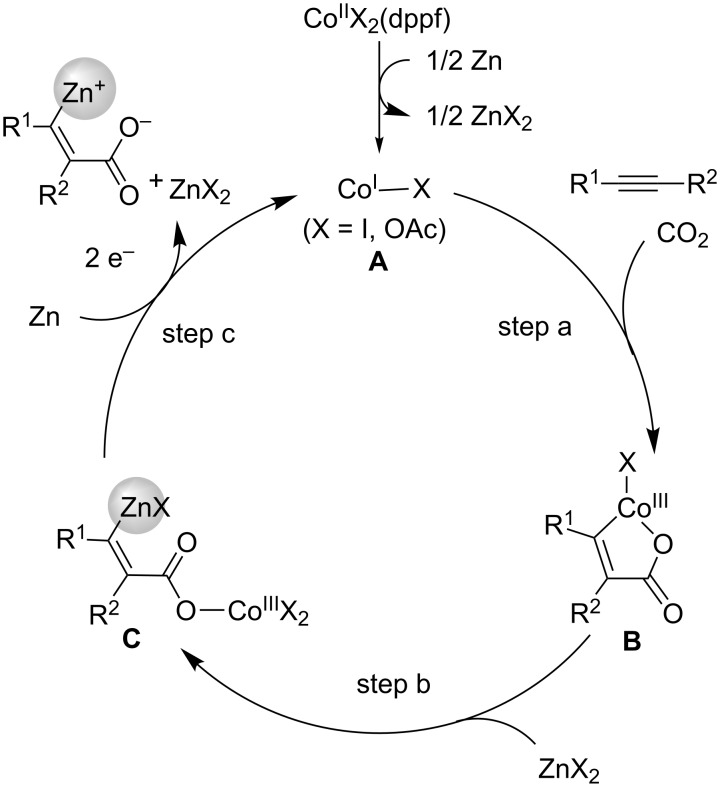
Plausible reaction mechanism for the Co-catalyzed carboxyzincation of alkynes **16**.

A possible reaction mechanism for the carboxyzincation reaction is displayed in Scheme 17. First, the Co(II) precursor is reduced to Co(I) (**A**) in the presence of metallic Zn. The oxidative cyclization of **A** with alkyne **16** and CO_2_ affords cobaltacycle **B** (step a). Next, the transmetalation between **B** and the Zn(II) species occurs, which affords the alkenylzinc intermediate **C** (step b) [41], which is then reduced with Zn powder, thereby giving the carboxyzincated product and regenerating Co(I) species **A** (step c).

**Scheme 18:**
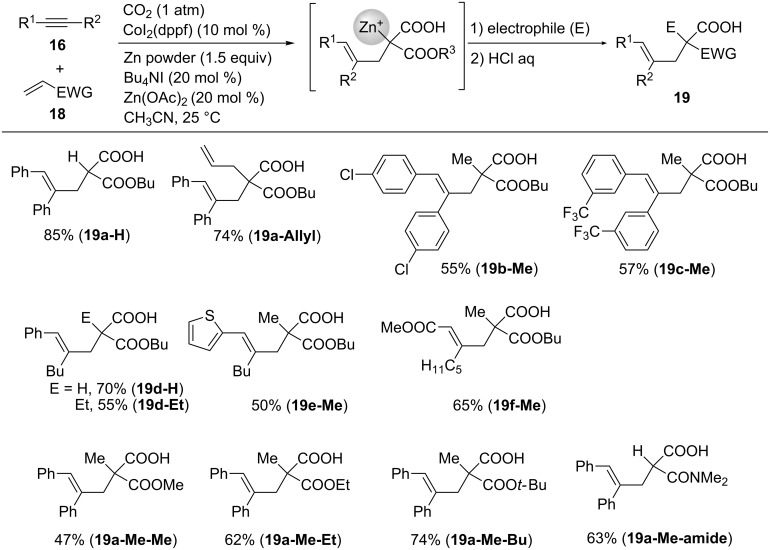
Co-catalyzed four-component coupling of alkynes **16**, acrylates **18**, CO_2_, and zinc.

We also achieved the four-component coupling of alkynes **16**, acrylates **18**, CO_2_, and Zn metal, as depicted in Scheme 18 [40]. As a model reaction, the reaction using diphenylacetylene (**16h**), butyl acrylate (**18a**), CO_2_, and Zn was performed. After treatment with H_2_O and allyl bromide, the corresponding products **19a-H** and **19a-Allyl** were obtained in high yields. Chloro and trifluoromethyl functionalities were well tolerated under the reaction conditions, and **19b-Me** and **19c-Me** were obtained in 55% and 57% yields, respectively. The reaction of unsymmetrical 1-phenyl-1-hexyne with **18a** afforded **19d-H** and **19d-Et** regioselectively. In addition, an alkyne with a thiophene ring regioselectively produced the desired product **19e-Me** after methylation with MeI. It is noteworthy that an alkynoate was also converted into the corresponding product **19f-Me** in good yield. Methyl, ethyl, and *tert*-butyl acrylates **18b**, **18c**, and **18d**, respectively, also afforded the corresponding products. The product of the reaction with acrylamide **18e** was also obtained.

**Scheme 19:**
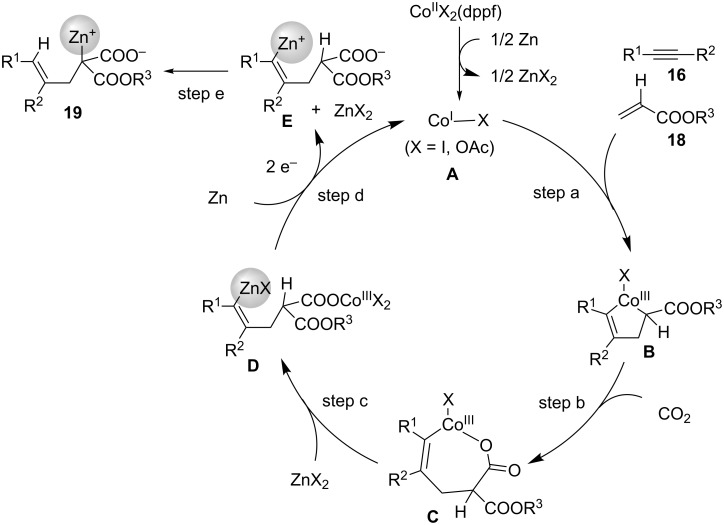
Proposed reaction mechanism for the Co-catalyzed four-component coupling.

Scheme 19 shows a plausible reaction mechanism for this four-component coupling reaction. In a similar manner to that described for the carboxyzincation, the reduction of the Co(II) precursor to Co(I) species **A** in the presence of Zn metal activates the catalytic cycle. Next, the oxidative cyclization of **A**, alkyne **16**, and acrylate **18** proceeds regioselectively, and cobaltacycle **B** is formed (step a) [42]. Then, the insertion of CO_2_ into the Co–C(sp^3^) bond occurs, and the seven-membered Co intermediate **C** is obtained (step b). The transmetalation of **C** with the Zn(II) species proceeds then to afford the alkenylzinc species **D** (step c). The subsequent two-electron reduction of **D** with Zn metal occurs, and the alkenylzinc intermediate **E** is subsequently obtained, along with the regeneration of Co(I) species **A** (step d). After 1,4-migration of zinc in **E**, product **19** is obtained (step e).

#### Visible-light-driven hydrocarboxylation of alkynes

The Use of photoenergy to organic synthesis is of importance, since the highly reactive intermediate can be generated by photochemical reaction such as electron transfer and energy transfer [43–45]. Among them, light-energy-driven CO_2_ fixation reactions via C–C bond formation are promising in terms of mimicking photosynthesis. In 2015, Murakami et al. found the direct carboxylation reaction with CO_2_ under photo-irradiation reaction conditions [46]. Jamison et al. also reported the synthesis of α-amino acid derivatives using amine and CO_2_ [47]. Iwasawa disclosed the Pd-catalyzed carboxylation of aryl halides using CO_2_ in the presence of an Ir photo-redox catalyst under visible-light irradiation conditions [48].

**Scheme 20:**
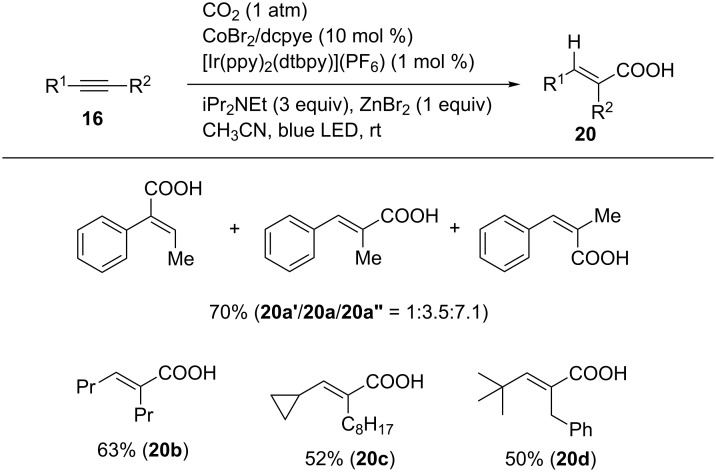
Visible-light-driven hydrocarboxylation of alkynes.

Zhao and Wu reported the visible-light-driven hydrocarboxylation of alkynes in the presence of a Co catalyst [49]. The reaction of alkynes was carried out using CoBr_2_/dcype (dcype = bis(dicyclohexylphosphino)ethane) as catalysts in the presence of [Ir(ppy)(dtbpy)](PF_6_) and iPr_2_NEt as photoredox catalyst and a sacrificial reagent, respectively, in acetonitrile under an atmospheric pressure of CO_2_ (Scheme 20). 1-Phenyl-1-propyne (**16n**) afforded hydrocarboxylated products as a mixture of regio- and stereoisomers. 4-Octyne (**16b**) afforded the product **20b** in good yield. Other unsymmetrical internal alkynes were converted to the corresponding products regioselectively.

**Scheme 21:**
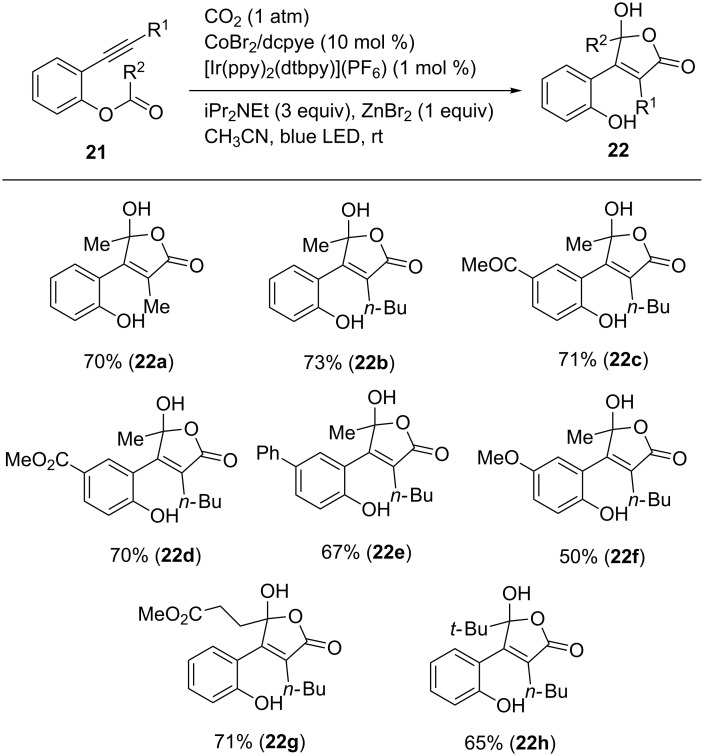
Visible-light-driven synthesis of γ-hydroxybutenolides from *ortho*-ester-substituted aryl alkynes.

The same protocol could be expanded to the synthesis of γ-hydroxybutenolides by using arylalkynes bearing *ortho*-esters of the aromatic ring (Scheme 21) [49]. Various alkynes **21** were converted to the corresponding products in moderate-to-good yields. Notably, ketone (**22c**) and ester (**22d**,**g**) functionalities were tolerated in the reaction. A bulky ester moiety took part in the reaction and the corresponding product **22h** was obtained in good yield.

**Scheme 22:**
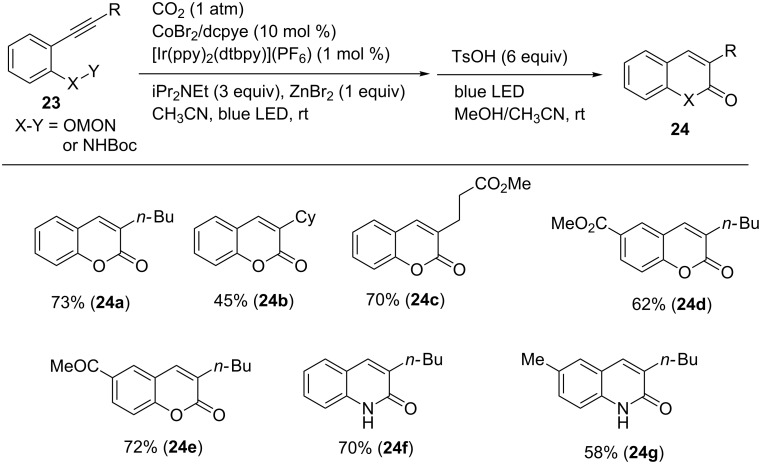
One-pot synthesis of coumarines and 2-quinolones via hydrocarboxylation/alkyne isomerization/cyclization.

Furthermore, the same group discovered the one-pot synthesis of coumarin derivatives via hydrocarboxylation/alkene isomerization/cyclization reactions (Scheme 22) [49]. A key of the sequential reactions is a use of aromatic alkynes bearing a momo-protected hydroxy group at the *ortho* position on the aromatic ring (**23**). The corresponding coumarin derivatives were obtained in moderate-to-good yields. Notably, ketone (**24e**), ester (**24d**) moieties were tolerated in the reaction. In addition, 2-quinolones (**24f** and **24g**) were obtained using alkynes bearing a Boc protected carbamate in place of the MOM protected ether.

**Scheme 23:**
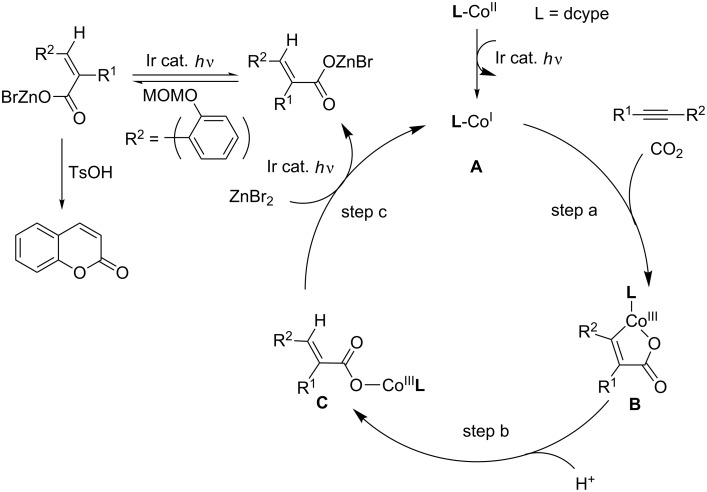
Proposed reaction mechanism for the Co-catalyzed carboxylative cyclization of *ortho*-substituted aromatic alkynes.

Scheme 23 shows a plausible reaction mechanism for these reactions. First, the Co(II) precursor is reduced to Co(I) **A** by the aid of an Ir photoredox catalyst and an amine under irradiation. The oxidative cyclization of **A** with **23** and CO_2_ affords cobaltacycle **B** (step a). Next, the protonation of **B** affords an intermediate **C** (step b). Finally, two-electron reduction of Co(III) in **C** occurs and Co(I) species **A** regenerates (step c). ZnBr_2_ may facilitate the step. Under the irradiation conditions, an *E*-isomer with aryl moiety can undergo a reversible isomerization to form the corresponding Z-isomer. Acid-mediated cyclization affords a coumarin derivative.

### Rhodium catalysts

#### Carboxylation of aryl and alkenylboronic esters

Aryl and alkenylboronic acids or their esters are of interest in organic synthesis because they are commonly used for C–C bond-forming reactions such as Pd-catalyzed Suzuki–Miyaura coupling reactions [50–53].

**Scheme 24:**
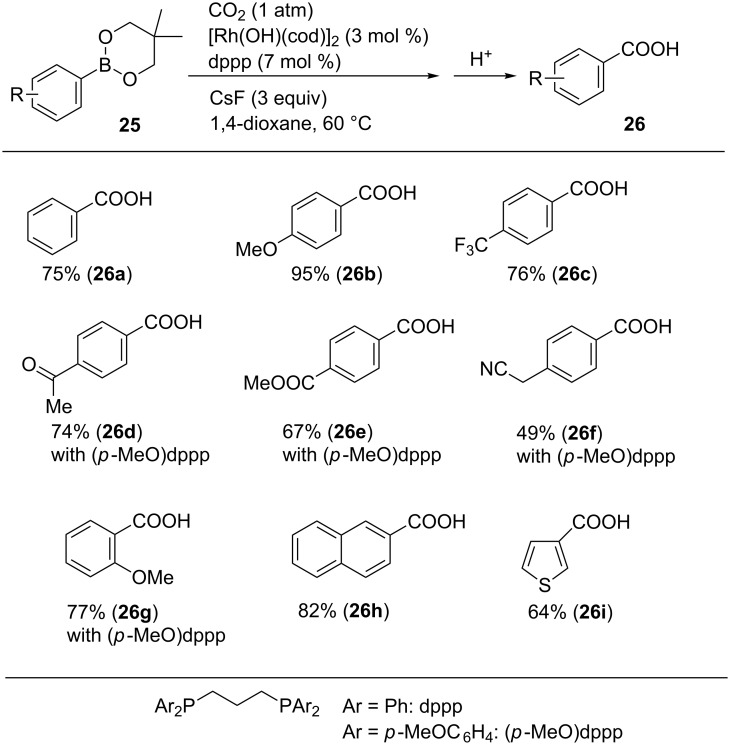
Rh-catalyzed carboxylation of arylboronic esters **25**.

Iwasawa et al. reported the Rh-catalyzed carboxylation of arylboronic esters using CO_2_ (Scheme 24) [54]. The reaction of **25a** was performed using a catalytic amount of [Rh(OH)(cod)]_2_ and 1,3-bis(diphenylphosphino)propane (dppp) in the presence of CsF as a base in 1,4-dioxane at 60 °C. Under these reaction conditions, the desired carboxylated product **26a** was obtained in 75% yield. A variety of arylboronic esters (**25b**–**i**) were converted into the corresponding carboxylic acids **26b**–**i** in good-to-high yields. It is noteworthy that ketone, ester, and nitrile functionalities in **26d**, **26e**, and **26f**, respectively, were tolerated in the reaction. Sterically hindered substrates could be subjected to the reaction, and **26g** was obtained from **25g**. Moreover, a substrate having a heteroaromatic ring (**25i**) was converted into its corresponding carboxylic acid **26i**.

**Scheme 25:**
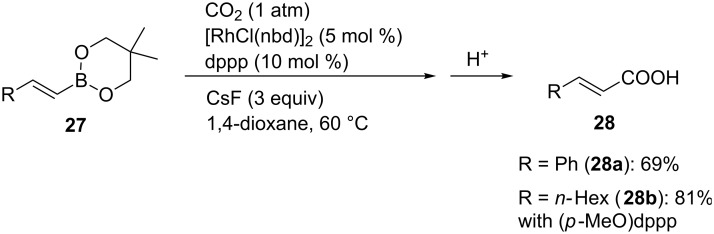
Rh-catalyzed carboxylation of alkenylboronic esters **27**.

Alkenylboronic esters **27** were also converted into the corresponding α,β-unsaturated carboxylic acids **28** using [RhCl(nbd)]_2_ (nbd = norbornadiene) as a catalytic precursor (Scheme 25) [54]. When an alkyl-substituted substrate was examined, the *p*-methoxy-substituted dppp derivative was found to be the suitable ligand.

**Scheme 26:**
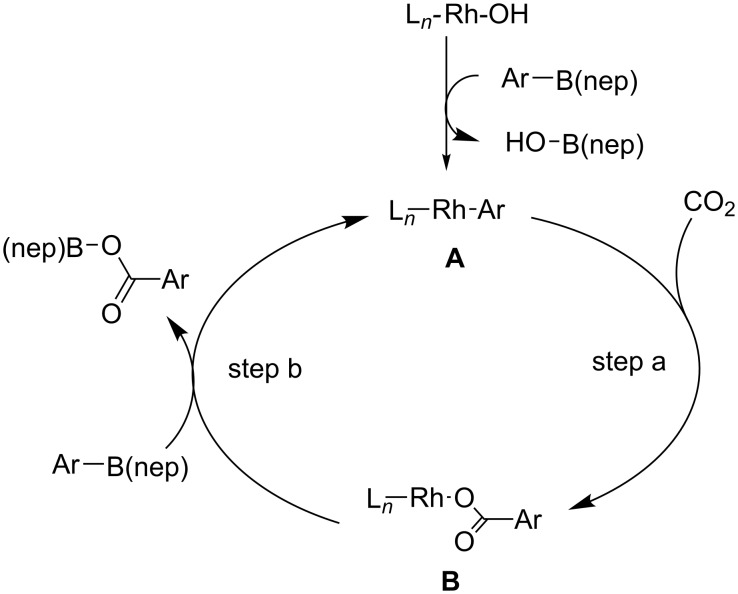
Plausible reaction mechanism for the Rh-catalyzed carboxylation of arylboronic esters **25**.

For this transformation, the reaction mechanism depicted in Scheme 26 was proposed. The catalytic cycle is activated by the generation of aryl-Rh intermediate **A** from the reaction of the Rh(I) species with the corresponding arylboronic ester. Next, the reaction of **A** with CO_2_ proceeds with the concomitant generation of the corresponding carboxylate Rh species **B** (step a). Finally, transmetalation between **B** and the arylboronic ester affords the product, along with the aryl-Rh intermediate **A** (step b)

After this contribution, the Cu-catalyzed carboxylation of aryl and alkenylboronic esters was independently reported by the groups of Iwasawa and How [55–56].

#### Direct C(sp^2^)–H bond carboxylation

As described above, C–H carboxylations with CO_2_, particularly C(sp^2^)–H carboxylation reactions, have attracted much research interest. As a consequence, Nolan [57] and Hou [58] independently reported Cu-catalyzed carboxylations using heteroarenes as substrates, which occur at the relatively acidic C–H bond.

**Scheme 27:**
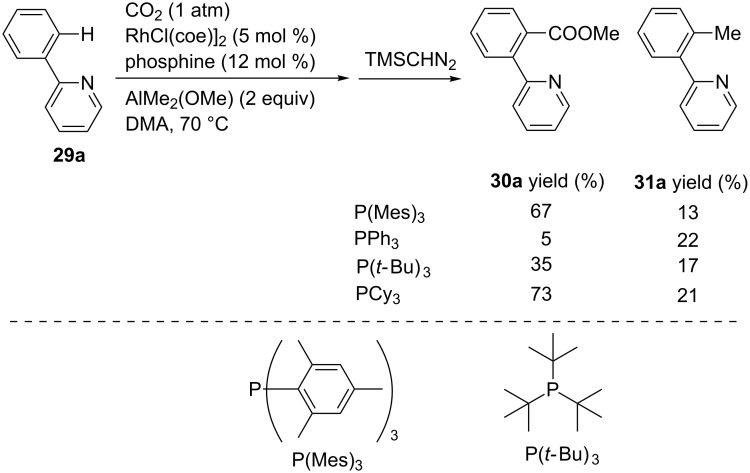
Ligand effect on the Rh-catalyzed carboxylation of 2-phenylpyridine **29a**.

Regarding Rh as the catalyst, Iwasawa et al. first reported a rhodium-catalyzed chelation-assisted C(sp^2^)–H carboxylation using methylaluminum as a reducing reagent (Scheme 27) [59]. Subsequently, the reaction of 2-phenylpyridine (**29a**) was performed using AlMe_2_(OMe) in DMA at 70 °C. Employing [RhCl(coe)]_2_ (coe = cyclooctene) and P(Mes)_3_ ((P(Mes)_3_ = tris(2,4,6-trimethylphenyl)phosphine) as the catalyst, the carboxylated product **30a** was obtained in 67% yield, along with the formation of a methylated byproduct **31a**. Other phosphine ligands such as PPh_3_, P(*t*-Bu)_3_, and PCy_3_ afforded the product in low-to-good yields.

**Scheme 28:**
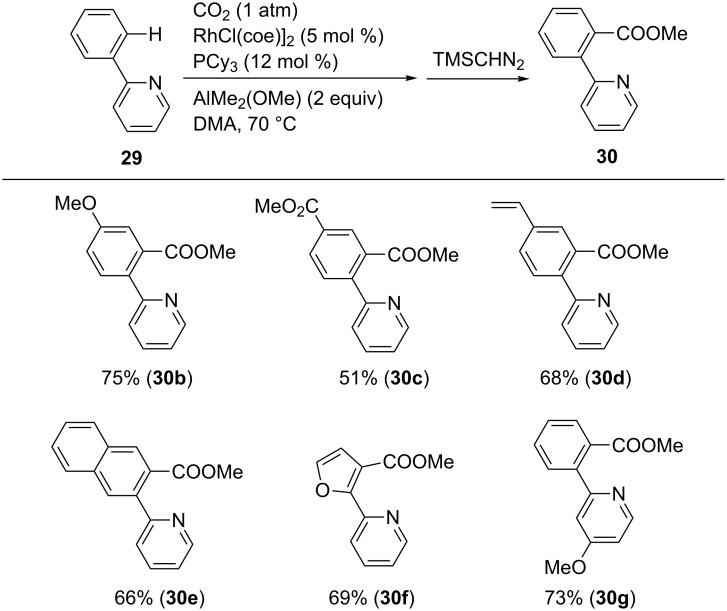
Rh-catalyzed chelation-assisted C(sp^2^)–H bond carboxylation with CO_2_.

Under the optimal reaction conditions using PCy_3_ as the ligand, various 2-pyridylarenes **29** were converted into the corresponding products **30** (Scheme 28). Substrates bearing either electron-donating or electron-withdrawing substituents at the aryl ring afforded the corresponding products. Interestingly, a terminal alkenyl group remained intact after the reaction (**30d**). Furthermore, substrates bearing naphthyl or furyl rings were carboxylated, and the corresponding products, **30e** and **30f**, respectively, were obtained in good yields.

**Scheme 29:**
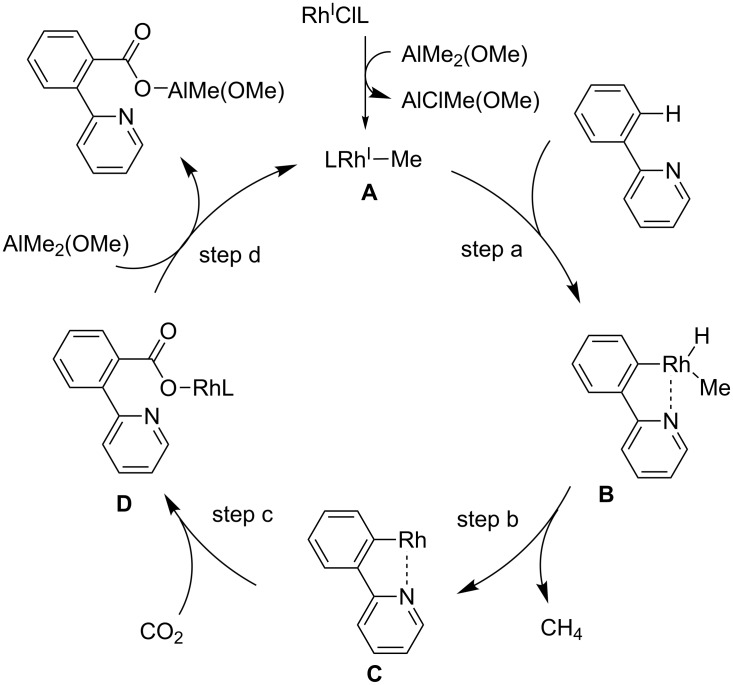
Reaction mechanism for the Rh-catalyzed C(sp^2^)–H carboxylation of 2-pyridylarenes **29**.

A plausible reaction mechanism for this Rh-catalyzed chelation-assisted C(sp^2^)–H bond carboxylation is shown in Scheme 29. First, a low-valent methyl–Rh(I) species **A** is generated by transmetalation. Secondly, a pyridine ring in the substrate coordinates to the Rh center, which prompts the cleavage of the adjacent C–H bond, affording Rh(III) species **B** (step a). Subsequently, the reductive elimination of methane from **B** affords the low-valent Rh(I) species **C**. Then, C–C bond formation with CO_2_ proceeds, and Rh carboxylate **D** is formed. Finally, the carboxylated product is obtained by the transmetalation between **D** and AlMe_2_(OMe), and methyl–Rh(I) **A** is regenerated.

**Scheme 30:**
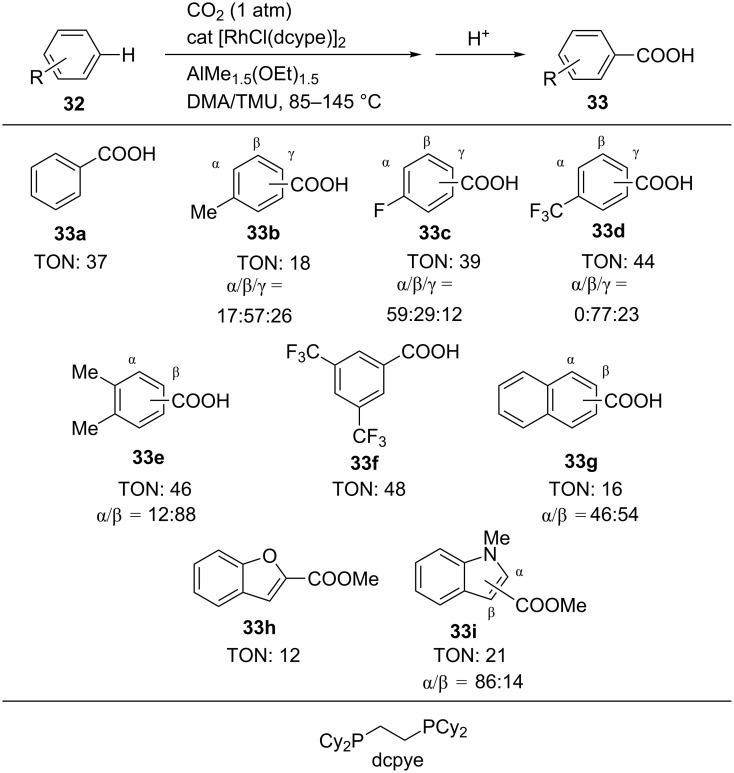
Carboxylation of C(sp^2^)–H bond with CO_2_.

Later, Iwasawa et al. achieved the Rh-catalyzed direct carboxylation of arenes without any directing group (Scheme 30) [60–61]. The reactions proceeded using a catalytic amount of Rh complex bearing dcype (dcype = 1,2-bis(dicyclohexylphosphino)ethane) as the ligand and AlMe_2_(OEt) as a reducing agent in a mixture of DMA and 1,1,3,3-tetramethylurea (TMU) as a solvent. Under the reaction conditions, benzene (**32a**) was converted into benzoic acid (**33a**, TON: 37) at 85 °C. The monosubstituted arenes such as toluene (**32b**), fluorobenzene (**32c**), and trifluoromethylbenzene (**32d**) afforded the corresponding carboxylic acids **33b, 33c**, and **33d** in good TON. *o*-Xylene yielded its corresponding mixture of carboxylic acids **33e**. When 1,3-bis(trifluoromethyl)benzene (**32f**) was used as the substrate at 145 °C, the corresponding carboxylic acid **33f** was site-selectively obtained in good TON. Benzofuran (**32h**) and indole (**32i**) also gave the carboxylic acids, which were isolated as their methyl esters.

**Scheme 31:**
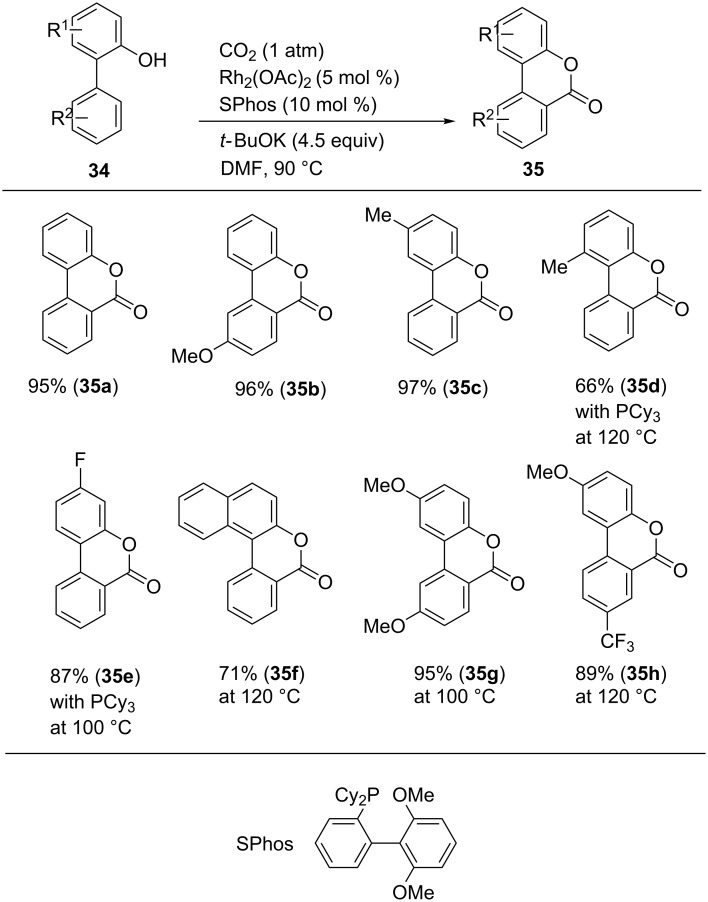
Carboxylation of C(sp^2^)–H bond with CO_2_.

Li and co-workers reported the Rh-catalyzed site-selective C(sp^2^)–H carboxylation reaction using 2-arylphenols as the substrates (Scheme 31) [62]. The desired reactions proceeded using a Rh_2_(OAc)_4_/SPhos catalyst system and *t-*BuOK as a base in DMF. Under the optimal reaction conditions, the reaction with 2-phenylphenol (**34a**) afforded dibenzopyranone (**35a**) in 95% yield. Other substituted 2-arylphenol derivatives (**34b–h**) were converted to the corresponding dibenzopyranones (**35b–h**) in good-to-high yields. Notably, sterically hindered substrates (**34d** and **34f**) were allowed by elevating the reaction temperature.

**Scheme 32:**
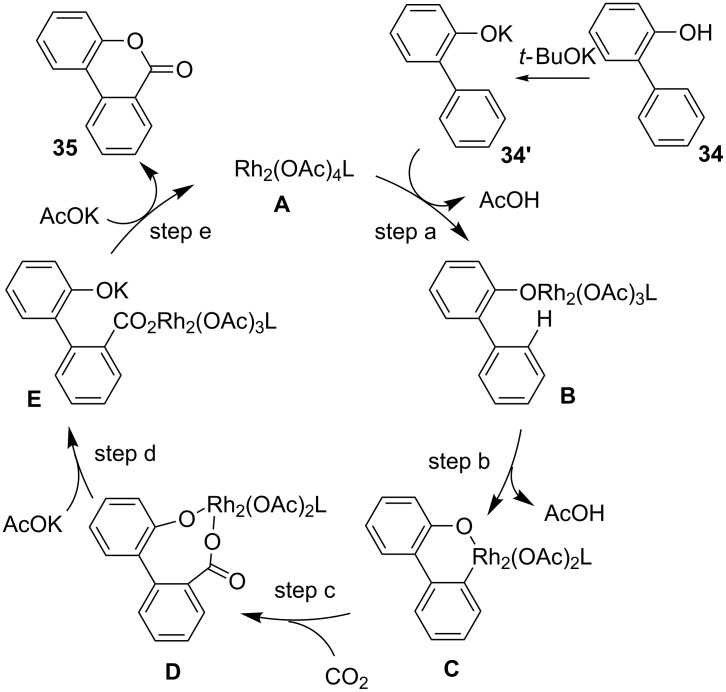
Reaction mechanism for the Rh-catalyzed C(sp^2^)–H carboxylation of 2-arylphenols **34**.

A plausible reaction mechanism is shown in Scheme 32. First, a phenoxide **34’** generated by the reaction of 2-arylphenol with *t-*BuOK reacts with a Rh complex **A** to generate a Rh complex **B** (step a). Then, chelation-assisted C–H bond activation proceeds to generate a rhodacycle **C** (step b). The reaction of **C** with CO_2_ affords an eight-membered rhodacycle intermediate **D** (step c). Next, **D** is converted to the corresponding rhodium complex **E** by ligand exchange with KOAc (step d). Possibly another ligand exchange between **E** and KOAc regenerates the Rh complex **A** (step e). The desired product (**35**) is obtained after lactonization.

#### Hydrocarboxylation of arylalkenes

**Scheme 33:**
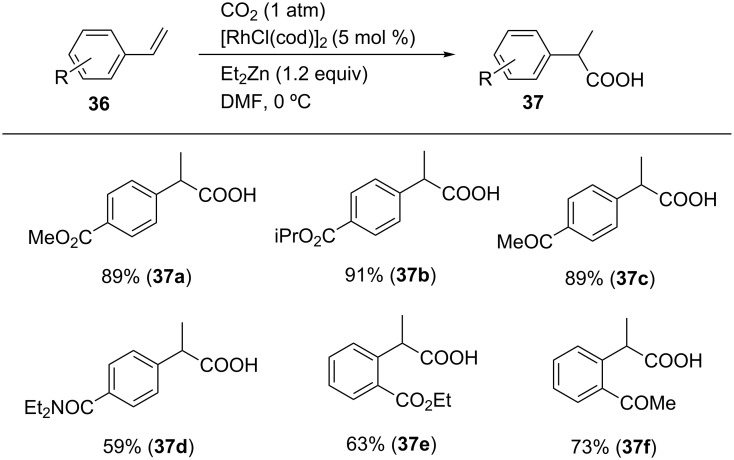
Hydrocarboxylation of styrene derivatives with CO_2_.

Hydrocarboxylation is an essential carboxylation reaction. To date, transition-metal-catalyzed hydrocarboxylation reactions using alkynes [63–66], alkenes [67–68], allenes [69–71] and 1,3-dienes [72–73] have been reported. In this regard, Mikami et al. reported the Rh-catalyzed hydrocarboxylation of styrene derivatives depicted in Scheme 33 [74]. The desired reaction proceeded using [RhCl(cod)]_2_ as a catalyst and Et_2_Zn as a reducing agent in DMF at 0 °C. As a result, diverse styrene derivatives **36a**–**f** bearing an electron-withdrawing group were converted into their corresponding carboxylic acids **37a**–**f** in moderate-to-high yields. Notably, the ester, ketone, and amide functionalities of **37a**, **37c**, and **37d**, respectively, were tolerated in the reaction. However, substrates such as 4-methoxystyrene or styrene did not yield the desired products.

**Scheme 34:**
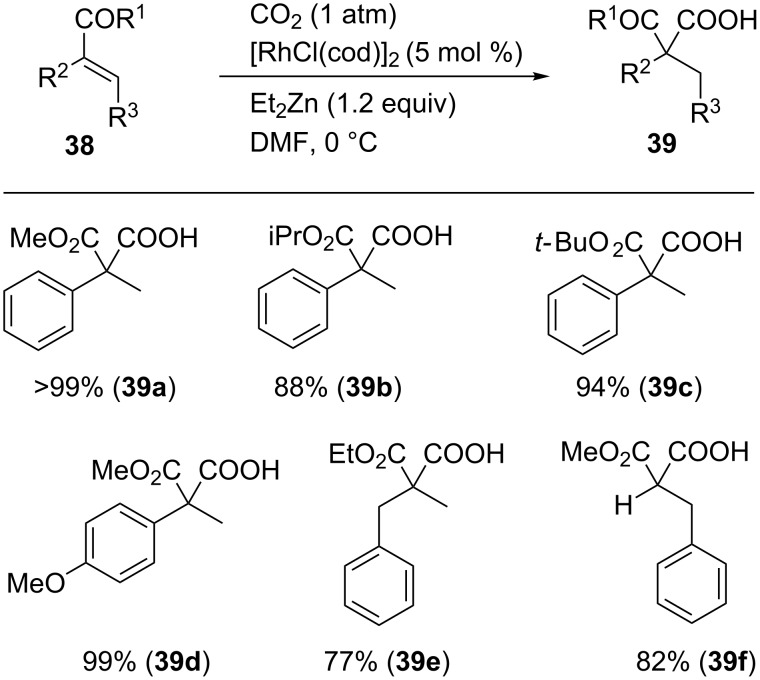
Hydrocarboxylation of α,β-unsaturated esters with CO_2_.

The same Rh-catalytic system proved to be applicable to the carboxylation of α,β-unsaturated esters **38** (Scheme 34). A variety of substrates **38a**–**f**, including those containing an electron-donating substituent or benzyl-substituted esters, were converted into their corresponding products **39a**–**f** in good-to-high yields.

**Scheme 35:**
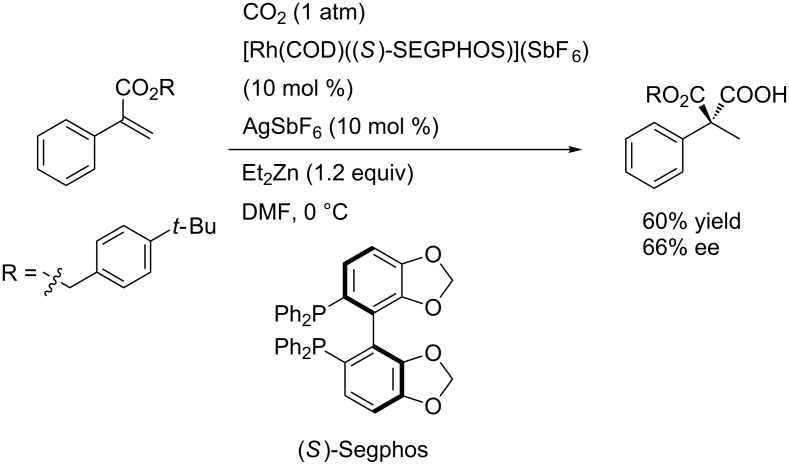
Asymmetric hydrocarboxylation of α,β-unsaturated esters with CO_2_.

Notably, the asymmetric hydrocarboxylation was archived by using a chiral bisphosphine as a ligand (Scheme 35).

**Scheme 36:**
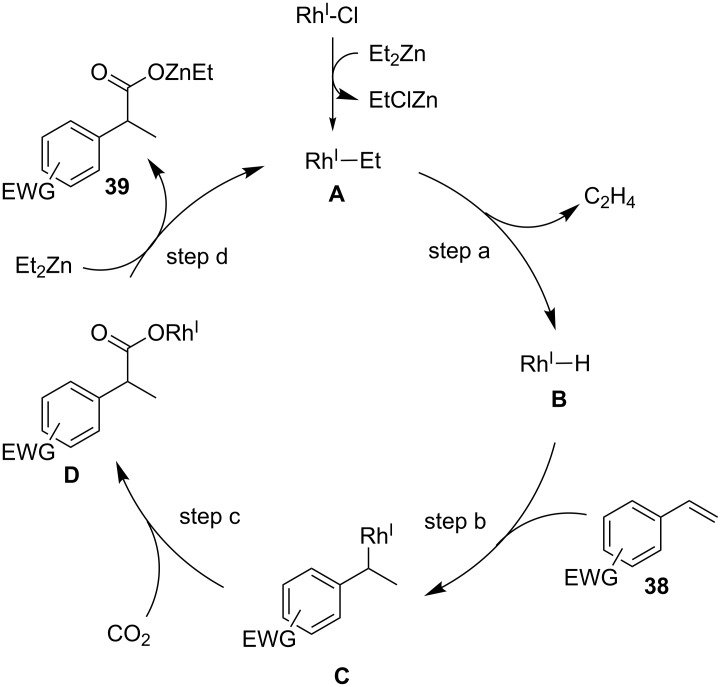
Proposed reaction mechanism for the Rh-catalyzed hydrocarboxylation of C–C double bonds with CO_2_.

Scheme 36 illustrates a plausible reaction mechanism for this transformation. First, transmetalation between the Rh(I) and Zn reagents generates ethyl–Rh(I) species **A**, from which β-hydrogen elimination occurs to yield the hydride-Rh intermediate **B** (step a). Subsequently, the hydrorhodation of the C–C double bond occurs, affording an alkyl-Rh(I) species **C** (step b). Then, C–C bond formation with CO_2_ proceeds to give Rh carboxylate **D** (step c) Finally, the carboxylated product is obtained by the transmetalation between **D** and Et_2_Zn, with the concomitant regeneration of ethyl–Rh(I) **A** (step d).

#### Visible light-driven hydrocarboxylation of alkenes

**Scheme 37:**
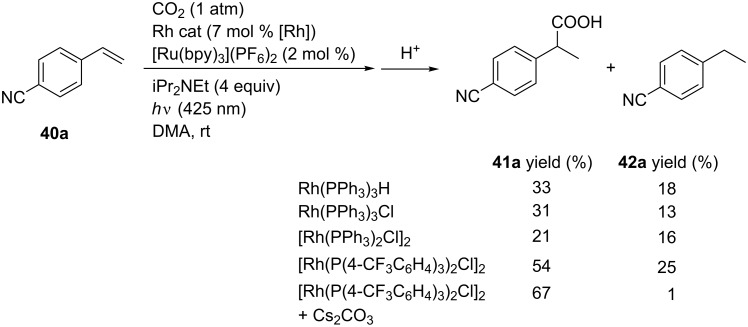
Visible-light-driven hydrocarboxylation with CO_2_.

Iwasawa et al. reported the Rh-catalyzed hydrocarboxylation of alkenes driven by visible-light irradiation conditions in the presence of a photoredox catalyst (Scheme 37) [75]. A model reaction using 4-cyanostyrene (**40a**) was carried out using iPrNEt_2_ as a sacrificial electron donor in the presence of [Ru(bpy)_3_](PF_6_)_2_ as a photoredox catalyst under visible-light irradiation (425 nm). Employing Rh(PPh_3_)_3_H as a catalyst, the desired hydrocarboxylated product **41a** was obtained in 33% yield along with the formation of reduced product **42a**. Rh(PPh_3_)_3_Cl and [Rh(PPh_3_)_2_Cl]_2_ were not efficient while a use of [Rh(P(4-CF_3_C_6_H_4_)_3_)_2_Cl]_2_ afforded **41a** in 54% yield. Finally, an addition of Cs_2_CO_3_ dramatically reduced the byproduct and **41a** was obtained in 67% yield.

**Scheme 38:**
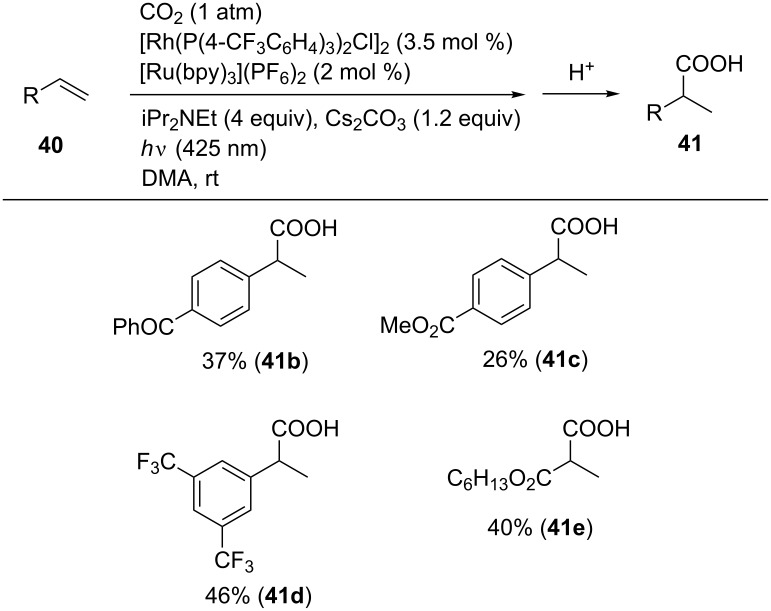
Visible-light-driven Rh-catalyzed hydrocarboxylation of C–C double bonds with CO_2_.

Under the optimal reaction conditions, several substrates were examined and the corresponding hydrocarboxylated products were obtained in moderate yields (Scheme 38).

#### [2 + 2 + 2] Cycloaddition of diynes with CO_2_

**Scheme 39:**
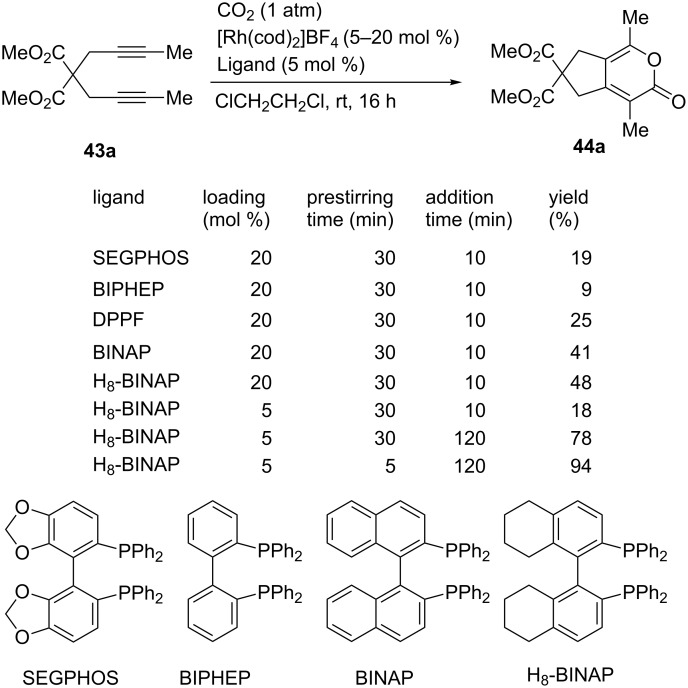
Optimization of reaction conditions on the Rh-catalyzed [2 + 2 + 2] cycloaddition of diyne **42a** and CO_2_.

The [2 + 2 + 2] cycloaddition of diynes with CO_2_ is an important reaction in the field of CO_2_ fixation. In these reactions, cyclic esters such as pyrones can be obtained, which are classically catalyzed by Ni complexes [76–78]. Tanaka et al. reported that a Rh complex with a suitable bidentate ligand was an efficient catalyst for the [2 + 2 + 2] cycloaddition reaction (Scheme 39) [79]. The reaction of **43a** was performed using 20 mol % [Rh(cod)_2_]BF_4_ and a bidentate phosphine in 1,2-dichloroethane at room temperature. Prior to the addition of **43a**, the mixture of [Rh(cod)_2_]BF_4_ and phosphine was stirred for 30 min. Then, **43a** was added dropwise to the mixture over 10 min, and the resulting reaction mixture was further stirred for 16 h. As ligands, SEGPHOS, BIPHEP, and DPPF were ineffective, but BINAP and H_8_-BINAP afforded the product **44a** in moderate yields. Notably, the addition of **43a** over 120 min improved the yield even at low catalyst loadings (5 mol %). A high (94%) yield was eventually obtained by reducing the prestirring time to 5 min.

**Scheme 40:**
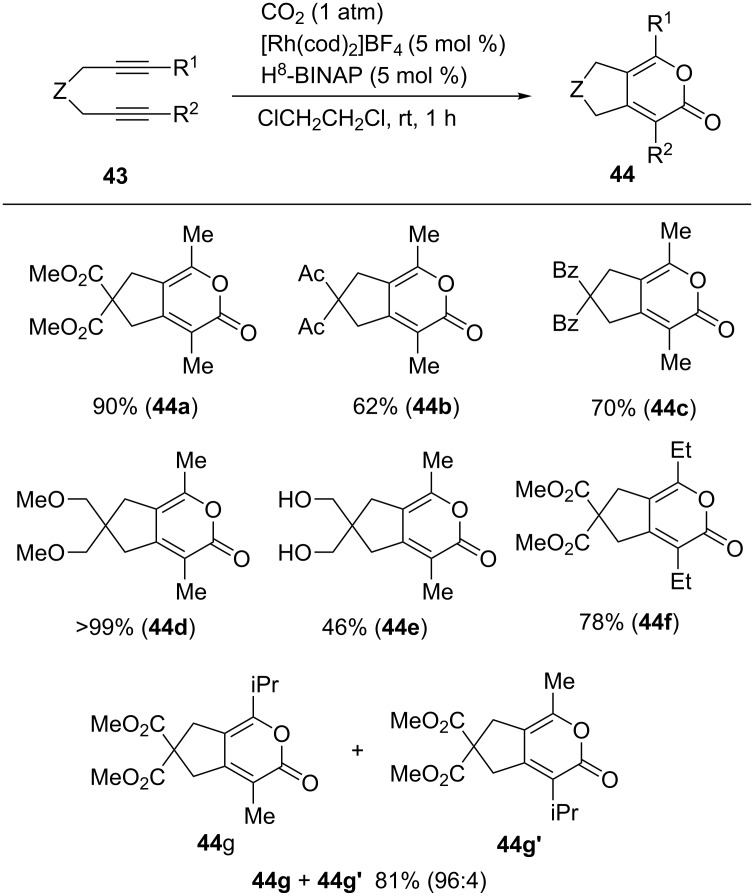
[2 + 2 + 2] Cycloaddition of diyne and CO_2_.

A variety of diynes having different tether units (**43a**–**g**) were converted into the corresponding pyrones **44a**–**g** in good-to-high yields within 1 h (Scheme 40). Ester, ketone, and hydroxy groups were tolerated in the reaction. In the case of an unsymmetrical diyne bearing methyl and isopropyl groups (**43g**), a mixture of regioisomers **44g** + **44g**′ was obtained in high yield with high regioselectivity.

**Scheme 41:**
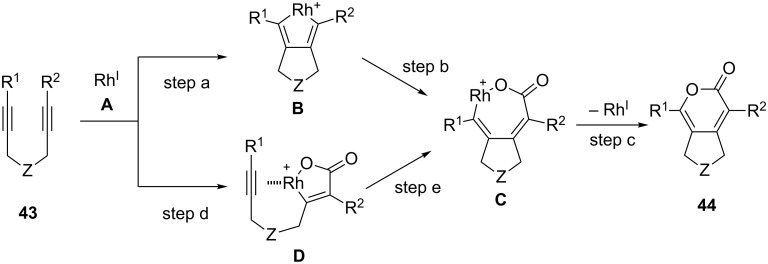
Proposed reaction pathways for the Rh-catalyzed [2 + 2 + 2] cycloaddition of diyne and CO_2_.

For this transformation, the reaction pathways depicted in Scheme 41 can be envisaged. The Rh(I) species **A** reacts with a diyne to afford rhodacycle **B** (step a). Then, the reaction of **B** with CO_2_ produces the seven-membered rhodium intermediate **C** (step b), from which reductive elimination occurs to yield its corresponding pyrone and the Rh(I) species **A** (step c). Alternatively, the oxidative cyclization of **A** proceeds as one of the C–C triple bonds of the diyne and CO_2_ react regioselectively, and rhodacycle **D** is subsequently formed (step d). Then, the insertion of the alkyne into the Rh–C bond occurs to give rhodacycle **C** (step e).

## Conclusion

In this review, the Co- and Rh-catalyzed transformation of CO_2_ via carbon–carbon bond-forming reactions is summarized. Co complexes can catalyze the carboxylation of propargyl acetates and alkenyl triflates. The cobalt-catalyzed reductive carboxylation of α,β-unsaturated nitriles and carboxyamides proceeds using Et_2_Zn. In addition, a cobalt complex proved to be an efficient catalyst in the allylic C(sp^3^)–H carboxylation. In the presence of zinc as the reagent, carboxyzincation and the four-component coupling reaction between alkyne, acrylates, CO_2_, and zinc occur efficiently. Rh complexes also catalyze the carboxylation of aryl and vinylboronic esters, the C(sp^2^)–H carboxylation of aromatic compounds, and the hydrocarboxylation of styrene derivatives. The Rh-catalyzed [2 + 2 + 2] cycloaddition of diynes and CO_2_ proceeds to afford pyrenes. Combinations of metals (cobalt or rhodium), substrates, and reducing agents can realize efficient carboxylation reactions using CO_2_ under mild reaction conditions. Furthermore, the development of novel carboxylation reactions using clean reducing agents such as non-metallic organic reductants such as amine, water, or hydrogen gas can be envisaged in the near future.
